# Real-time monitoring efficiency and toxicity of chemotherapy in patients with advanced lung cancer

**DOI:** 10.1186/s13148-015-0150-9

**Published:** 2015-11-05

**Authors:** Hong Wang, Bingfeng Zhang, Dan Chen, Wenying Xia, Jiexin Zhang, Fang Wang, Jian Xu, Yan Zhang, Meijuan Zhang, Lixia Zhang, Yachun Lu, Yan Geng, Peijun Huang, Puwen Huang, Hong Wang, Shiyang Pan

**Affiliations:** Department of Laboratory Medicine, The First Affiliated Hospital of Nanjing Medical University, Nanjing, 210029 China; National Key Clinical Department of Laboratory Medicine, Nanjing, 210029 China; Department of Oncology, The First Affiliated Hospital of Nanjing Medical University, Nanjing, 210029 China; Department of Respiratory Medicine, The First Affiliated Hospital of Nanjing Medical University, Nanjing, 210029 China

**Keywords:** Hypermethylation, Advanced lung cancer, Circulating DNA, *APC*, *RASSF1A*

## Abstract

**Background:**

The Response Evaluation Criteria in Solid Tumors (RECIST) guideline and Common Terminology Criteria for Adverse Events (CTCAE) criteria are used to assess chemotherapy efficiency and toxicity in patients with advanced lung cancer. However, no real-time, synchronous indicators that can evaluate chemotherapy outcomes are available. We wanted to evaluate tumor response and toxicity in advanced lung cancer chemotherapy by using a novel synchronous strategy.

**Results:**

We enrolled 316 patients with advanced lung cancer who were treated with cisplatin-based therapy and followed up them for 3 years. Plasma was obtained before and after every chemotherapy cycle. We quantitative assayed total plasma DNA and methylation of the *APC*/*RASSF1A* genes. Four parameters were assessed: methylation level before chemotherapy (meth_0 h_), methylation level 24 h after chemotherapy (meth_24 h_), total plasma DNA concentration before chemotherapy (DNA_0 h_), and total plasma DNA concentration 24 h after chemotherapy (DNA_24 h_). When meth_24 h_ > meth_0 h_ of at least one gene was used to predict tumor response, the correct prediction rate was 82.4 %. Additionally, patients for whom DNA_24 h_/DNA_0 h_ ≤ 2 had mild toxicities. Therefore, meth_24 h_ > meth_0 h_ and DNA_24 h_/DNA_0 h_ ≤ 2 were defined as criteria for better tumor response and fewer adverse events with a high correct prediction rate (84.7 %).

**Conclusions:**

Quantitative analysis of total plasma DNA and plasma *APC/RASSF1A* methylation provide a real-time synchronous rapid monitoring indicator for therapeutic outcomes of advanced lung cancer, which could be a reference or supplementary guidelines in evaluating chemotherapy effects.

**Electronic supplementary material:**

The online version of this article (doi:10.1186/s13148-015-0150-9) contains supplementary material, which is available to authorized users.

## Background

Patients with advanced lung cancer (ALC) are usually diagnosed after metastasis has occurred and therefore require chemotherapy. However, the high mortality rate and low 5-year survival rate are partly because of resistance to currently available chemotherapy regimens and ineffective monitoring methods for chemotherapy efficacy [1–9]. Several years ago, scholars suggested the notion of real-time apoptosis monitoring, the key point of which was the necessity to find a real-time monitor to provide the information about whether the chemotherapy was working [10]. The current Response Evaluation Criteria in Solid Tumors (RECIST) guideline, which is based on tumor size measured by chest X-ray or CT scan, and widely used tumor markers assay (such as carcinoembryonic antigen (CEA) and neuronal specific enolase (NSE)) are not sensitive enough to monitor chemotherapy effects in an individual patient because these markers require more than a month after chemotherapy to provide the information of interest. Consequently, no synchronous indicator that can rapidly evaluate chemotherapy outcomes is available.

Reportedly, total circulating DNA, derived from necrotic or apoptotic cells, can come from both normal cells and tumor cells. In a normal state, total circulating DNA is at a low level—mainly from a few apoptotic normal cells (usually lymphocytes and other nucleated cells) [11]. However, when both normal and tumor cells are killed by chemotherapy drugs, total circulating DNA will obviously increase; DNA from the normal cells will reflect the side effects of the drugs. Because aberrant genes hypermethylation usually occurs in neoplastic and abnormally differentiated cells [12–15], elevated gene methylation levels will be present if many cancer cells are killed by chemotherapy drugs (Additional file 1: Figure S1). Therefore, circulating tumor DNA with abnormal methylation patterns can be detected with a notable degree of specificity, even in the presence of excess DNA from normal cells. This was the start point for the present study to use total circulating DNA and methylation assays as indicators of the efficacy of chemotherapy in ALC patients.

Though many studies have been interested in the relationship between prognosis and methylation levels in tumor suppressor genes (TSGs), research that focuses on indicators that can simultaneously monitor not only tumor response but also toxicity after chemotherapy is rare. We designed this prospective research to present a detailed study on the quantitative dynamic alteration of *APC*/*RASSF1A* methylation levels before and after chemotherapy cycles and to combine assays of *APC*/*RASSF1A* methylation and total plasma DNA in the hope of providing a new strategy to monitor efficiency and toxicity of chemotherapy in ALC.

## Results

### Methylation levels increased 24 h after cisplatin administration in A549 cells and in tumor-bearing nude mice

To determine whether *APC* or *RASSF1A* gene promoters were methylated in A549 cells, we first assayed the methylation status of this cell line compared with H460 cells, which acted as a positive control. Ct values of *APC* and *RASSF1A* were 28.5 and 29.7 for H460 and 28.2 and 29.6 for A549, respectively, demonstrating positive methylation of APC and RASSF1A in A549 cells (Additional file 2: Figure S2).

**Fig. 1:**
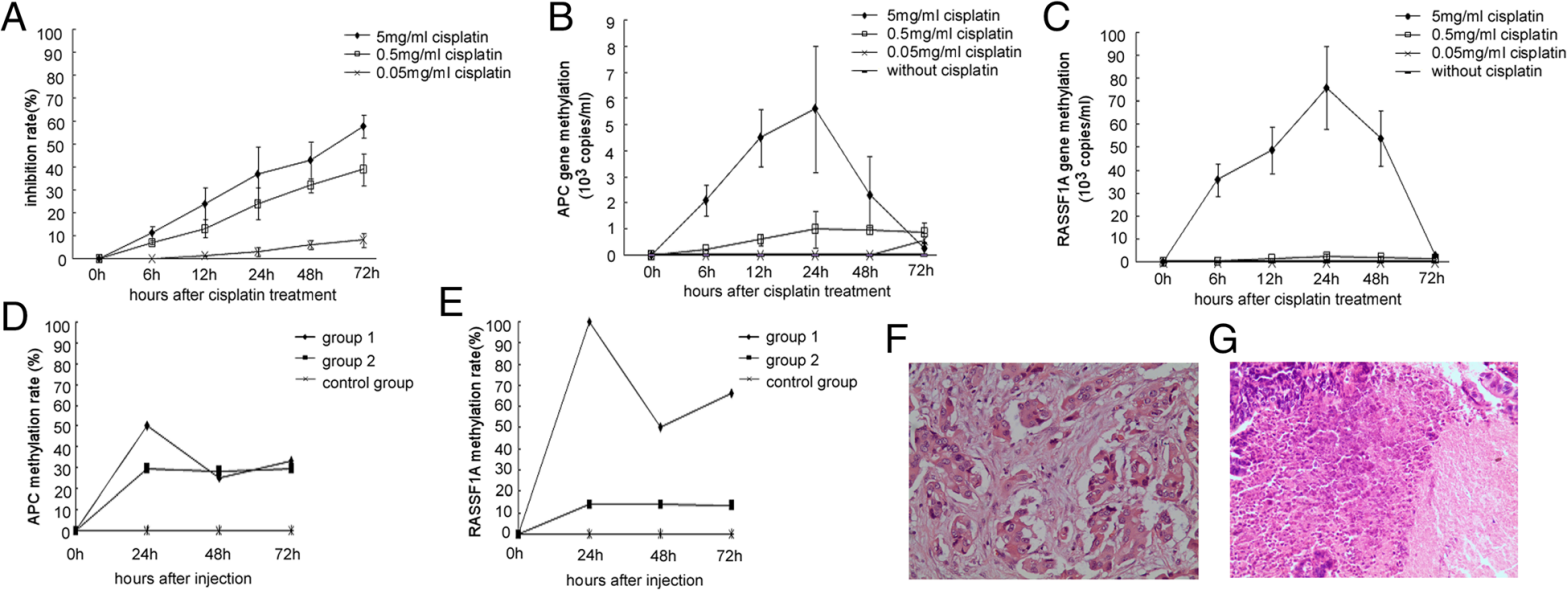
Methylation levels increased 24 h after cisplatin administration in A549 cells or in tumor-bearing nude mice. **a** MTT assay showed that A549 cells were sensitive to 5 mg/ml cisplatin. *Error bars* mean ± SD (*n* = 3). **b** The peak methylation level of *APC* in A549 cells was at 24 h after cisplatin treatment. **c** The peak methylation level of *RASSF1A* in A549 cells was at 24 h after cisplatin treatment. Methylation rates of **d** *APC* and **e** *RASSF1A* were highest at 24 h after cisplatin injection in tumor-bearing nude mice injected with NS and cisplatin (group 1). **f** Pathological biopsy of tumor tissues for tumor-bearing nude mice injected with NS (group 2). Most tumor cells in the tissues were alive at 72 h after injection (×200). **g** Biopsy of tumor tissues for mice of group 1. Tumor cells in the tissues had nearly all died at 72 h after injection (×200)

A549 cell supernatant was tested for its methylation status at 6, 12, 24, 48, and 72 h after treatment with different concentrations of cisplatin (Fig. 1). First, an 3-(4,5-dimethyl-2-thiazolyl)-2, 5-diphenyl-2H-tetrazolium bromide (MTT) assay (Fig. 1a) indicated that A549 cells were sensitive to 5 mg/ml cisplatin (inhibition rate >50 %). Second, under this optimal cisplatin dose (5 mg/ml), methylation of *APC* or *RASSF1A* peaked at 24 h (Fig. 1b, c).

For a better understanding of changes in methylation status of the two genes after cisplatin treatment, we used a tumor-bearing nude mouse model (Fig. 1d, e). The methylation rates of *APC* or *RASSF1A* in the plasma of tumor-bearing nude mice were highest at 24 h after injection. Biopsies showed that most tumor cells in the tissues of group 1 (treated with cisplatin) were dead, whereas tumor cells in group 2 (treated with normal saline) were still proliferating (Fig. 1f, g).

### Elevated methylation level after cisplatin-based chemotherapy was correlated with good tumor response in ALC patients of training study

**Fig. 2:**
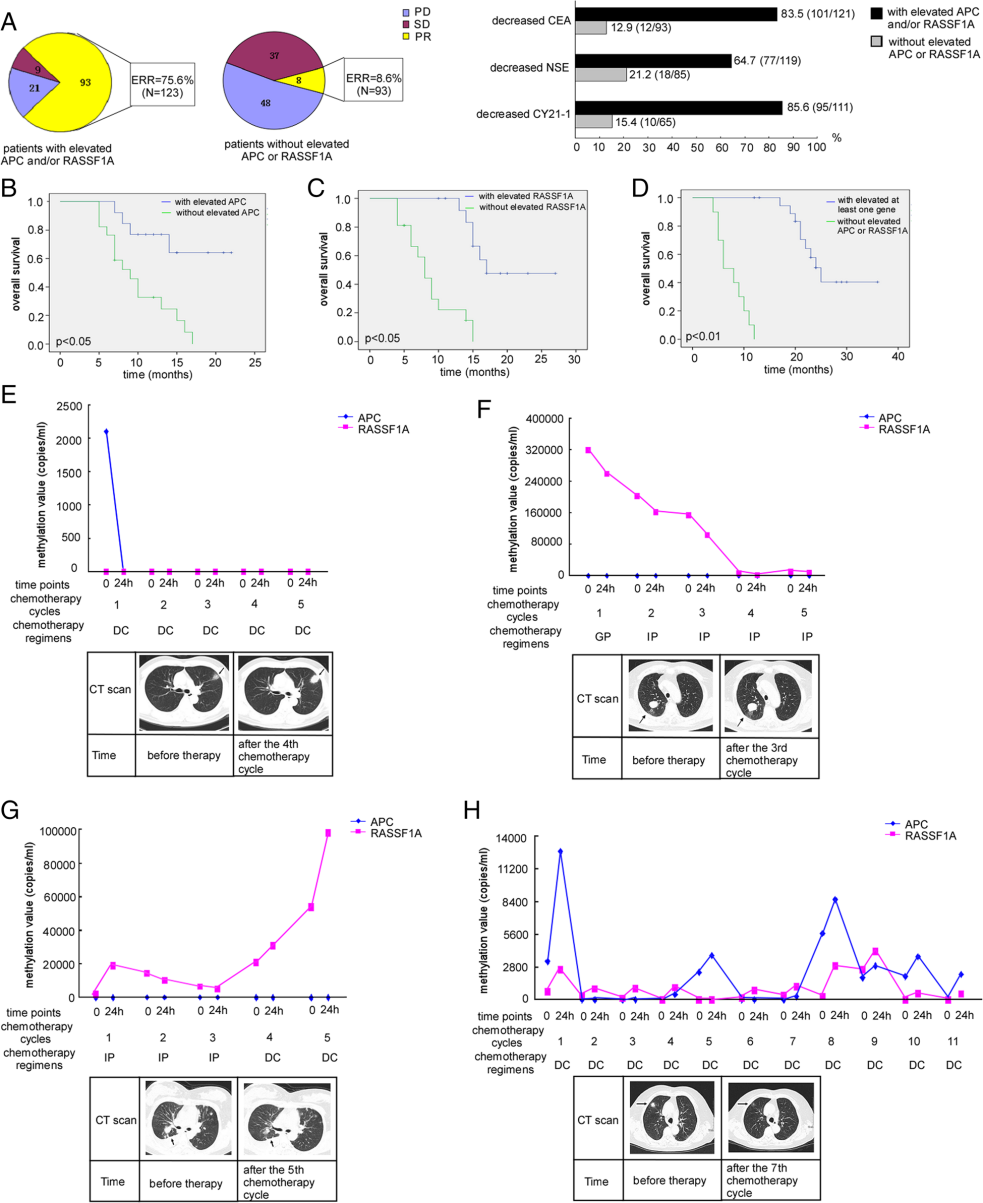
Elevated methylation level after cisplatin-based chemotherapy was correlated with good tumor response in ALC patients of training study. **a** Tumor response evaluation and tumor markers levels. In patients with elevated *APC* and/or *RASSF1A* methylation level, the efficient response rate (ERR) was higher; most cases had decreased tumor markers levels. **b** Kaplan–Meier curve for OS of patients with and without elevated *APC* methylation levels (median OS, 18 vs. 9 months, *P* < 0.05). **c** Kaplan–Meier curve for OS rates of patients with and without upregulated *RASSF1A* methylation levels (median OS, 20 vs. 8 months, *P* < 0.05). **d** Kaplan–Meier curves for OS rates of patients with elevated methylation levels of at least one gene and those without increased *APC* or *RASSF1A* methylation (median OS, 25 vs. 6 months, *P* < 0.01). **e**–**h** Methylation status and CT scan images of four representative patients. **e**, **f** Two patients died within 4 and 5 months of diagnosis, respectively. **g**, **h** Two patients remained alive during this study

Before chemotherapy, methylation frequencies of *APC* and/or *RASSF1A* were 48.9 % (46/94) in adenocarcinoma, 50.0 % (28/56) in squamous carcinoma, and 39.4 % (26/66) in other histological types. After two chemotherapy cycles, 123 patients had elevated *APC* and/or *RASSF1A* methylation (elevated gene methylation means the methylation level at 24 h [meth_24 h_] after the chemotherapy cycle was higher than that before chemotherapy [meth_0 h_], that is, meth_24 h_ > meth_0 h_). The efficient response rate (ERR) was 75.6 % (93/123). Among the 123 patients, there were 83.5 % (101/121), 64.7 % (77/119), and 85.6 % (95/111) cases whose CEA, NSE, and CY21-1 levels decreased. In the remaining 93 patients, who were without elevated *APC*/*RASSF1A* methylation, the ERR was 8.6 % (8/93), significantly lower than those with meth_24 h_ > meth_0 h_ of at least one gene (*P* < 0.01). CEA, NSE, and CY21-1 levels decreased in 12.9 % (12/93), 21.2 % (18/85), and 15.4 % (10/65) of these 93 patients (Fig. 2a). When we used meth_24 h_ > meth_0 h_ of at least one gene to predict tumor response, the correct prediction rate was 82.4 % [(93 + 37 + 48)/216].

We performed overall survival (OS) rate analyses according to increases in gene methylation levels after chemotherapy (Fig. 2b–d). The median survival was different between patients with methylation level elevation and those without elevation of *APC* or *RASSF1A* methylation.

The above results seemed to suggest that the meth_24 h_ > meth_0 h_ of at least one gene is correlated with good response to cisplatin-based chemotherapy in ALC patients. Large amounts of clinical data taken over a 3-year follow-up also confirmed this opinion (Fig. 2e–h).

### Elevated total plasma DNA after cisplatin-based chemotherapy was correlated with the adverse events grade in ALC patients of training study

**Fig. 3:**
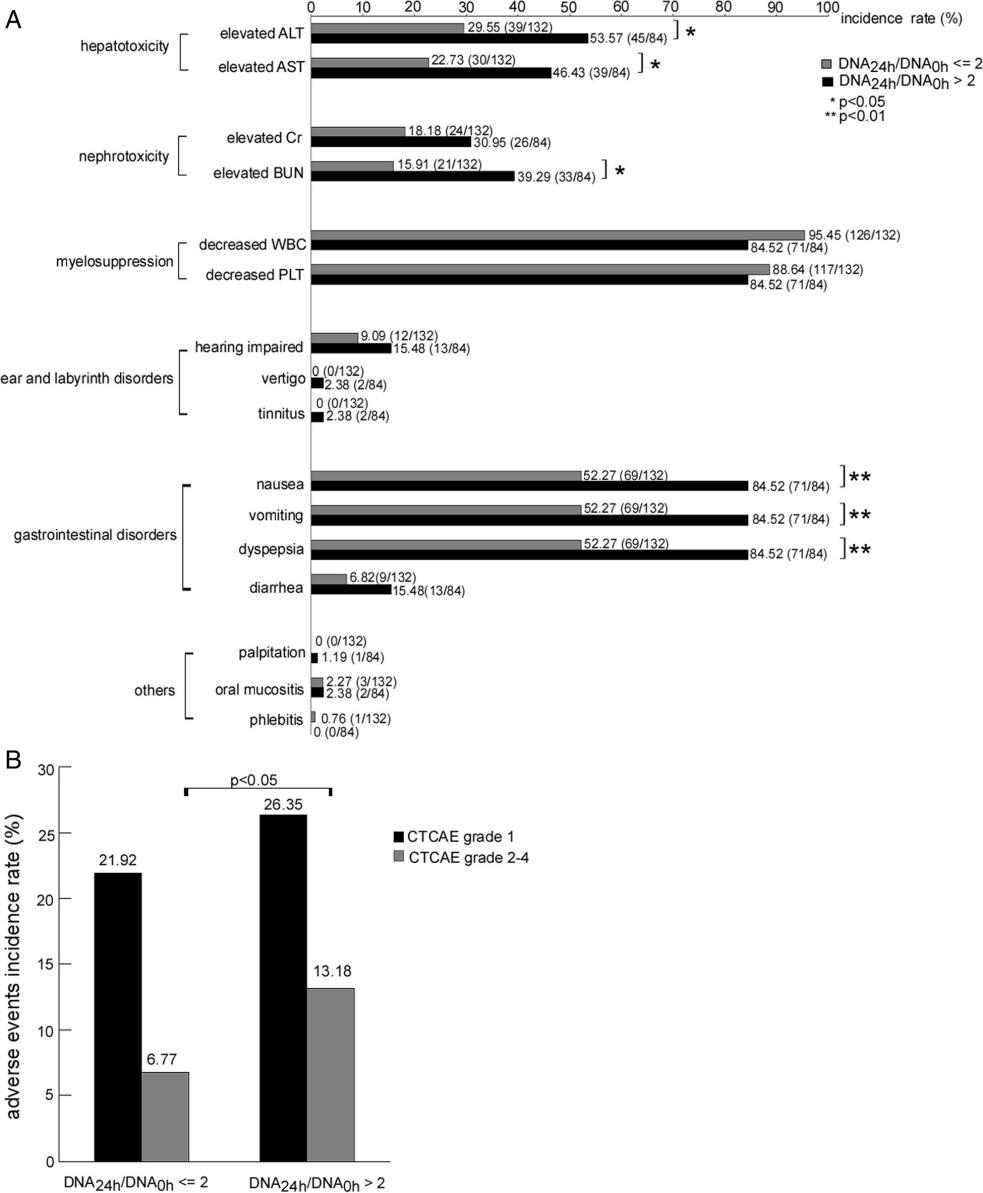
Elevated total plasma DNA after cisplatin-based chemotherapy was correlated with the adverse events grade in ALC patients of training study. **a** Toxicities of platinum-based regimens mainly occurred in 84 cases with DNA_24 h_/DNA_0 h_ > 2, especially hepatotoxicity, nephrotoxicity, and gastrointestinal symptoms. **b** The incidence of adverse events above grade 1 in the DNA_24 h_/DNA_0 h_ > 2 case group was significantly elevated compared with that in the DNA_24 h_/DNA_0 h_ ≤ 2 case group (13.18 vs. 6.77 %, *P* < 0.05)

Toxicities were evaluated after the first chemotherapy. From 216 paired plasma samples (before and 24 h after medication), we got the changes of total plasma DNA: total plasma DNA concentration at 24 h after medication (DNA_24 h_) was less than or equal to twofolds of that before medication (DNA_0 h_) in 132 cases (DNA_24 h_/DNA_0 h_ ≤ 2) and DNA_24 h_ was more than twofolds of DNA_0 h_ in 84 cases (DNA_24 h_/DNA_0 h_ > 2). Toxicities of platinum-based regimens, especially hepatotoxicity, nephrotoxicity, and gastrointestinal symptoms, mainly occurred in the 84 DNA_24 h_/DNA_0 h_ > 2 cases (Fig. 3a). The incidence of adverse events in the DNA_24 h_/DNA_0 h_ > 2 case group was higher than that in the DNA_24 h_/DNA_0 h_ ≤ 2 case group (39.53 vs. 28.69 %, *P* < 0.05). Especially, the incidence of adverse events above grade 1 in the DNA_24 h_/DNA_0 h_ > 2 case group was significantly elevated compared with that in the DNA_24 h_/DNA_0 h_ ≤ 2 case group (13.18 vs. 6.77 %, *P* < 0.05, Fig. 3b).

### Combined APC/RASSF1A methylation and total plasma DNA assay was consistent with clinical judgment in ALC patients of training study

**Fig. 4:**
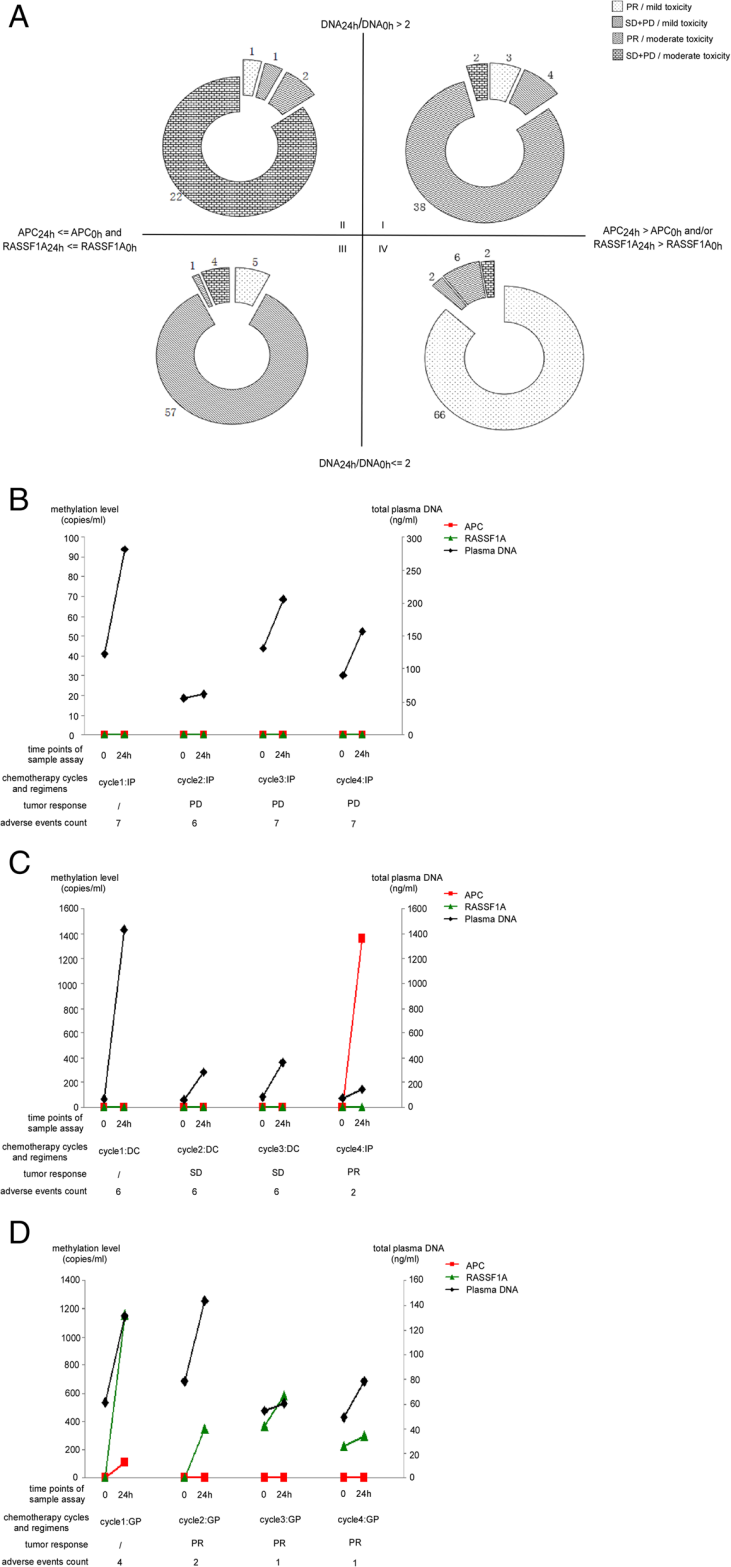
Combined APC/RASSF1A methylation and total plasma DNA assay was consistent with clinical judgment in ALC patients. **a** Patients with different methylation status and plasma DNA level had different tumor response and degrees of adverse events. The coincidence rates were  80.9, 84.6, 85.1, and 86.8 % in quadrants I to IV, respectively. **b**–**d** Methylation status, plasma DNA level, and some clinical information of three patients in the validation model. **b** A male patient died within 5 months of diagnosis. **c** A female patient had been alive for 36 months by the end of follow-up. **d** A patient had been alive nearly 24 months by the end of follow-up

After the second chemotherapy cycle, we found 123 patients who were meth_24 h_ > meth_0 h_ for *APC* and/or *RASSF1A*, 93 who were meth_24 h_ ≤ meth_0 h_ for both genes, 67 were DNA_24 h_/DNA_0 h_ > 2 patients, and 149 were DNA_24 h_/DNA_0 h_ ≤ 2 patients. Monitoring performance of combined *APC*/*RASSF1A* methylation and total plasma DNA assay, and the clinical judgment of tumor response and toxicity degree, is shown in Fig. 4a. Patients in quadrant I to quadrant IV were 47, 26, 67, and 76, respectively. In quadrant I, clinical judgment (tumor response and toxicity) was consistent with combined methylation and total plasma DNA assay in 38 cases. The coincidence rate was 80.9 % (38/47). Likewise, the coincidence rates were 84.6 % (22/26), 85.1 % (57/67), and 86.8 % (66/76) in quadrants II to IV, respectively. Therefore, the use of combined *APC*/*RASSF1A* methylation and total plasma DNA assay to predict chemotherapy outcome gave a correct prediction rate of 84.7 % [(22 + 57 + 38 + 66)/216].

### Further confirmation of the combined indicator in validation study

After the second chemotherapy cycle, 54 patients were judged to have complete response (CR)/partial response (PR) and mild adverse events; of these, 85.2 % (46/54) had “meth_24 h_ > meth_0 h_” of at least one gene and “DNA_24 h_/DNA_0 h_ ≤ 2”. In contrast, 28 patients had stable disease (SD)/progressive disease (PD) and moderate/severe adverse events, of whom 82.1 % (23/28) showed no *APC*/*RASSF1A* methylation elevation or had very high DNA_24 h._ Ten patients have CR/PR and moderate/severe adverse events, 80 % (8/10) had “meth_24 h_ > meth_0 h_” of at least one gene and DNA_24 h_/DNA_0 h_ > 2. Eight patients have SD/PD and mild adverse events, 75 % (6/8) showed no *APC*/*RASSF1A* methylation elevation and “DNA_24 h_/DNA_0 h_ ≤ 2”. Dynamic observation of cycles during follow-up was also analyzed. Figure 4b–d shows changes in total plasma DNA, *APC*/*RASSF1A* methylation, and clinical adverse events from three randomly chosen patients. This information also confirmed that patients with “meth_24 h_ > meth_0 h_” of at least one gene and “DNA_24 h_/DNA_0 h_ ≤ 2” had better tumor response and fewer adverse events.

## Discussion

Chemotherapy is usually the treatment of choice to improve the quality of life and prolong survival time of patients with advanced cancers. The response of malignant tumors to chemotherapy varies, and some clinical trials have shown that patients with advanced cancer who received personalized chemotherapeutic regimens or drug doses fared better than those treated with standard chemotherapy [16, 17], which has led to increased focus on personalized medicine [18]. However, existing detection means are not sensitive and timely enough to provide adequate information about chemosensitivity.

Cell death reportedly results in the release of DNA into the circulation, which peaks at 24 h and gradually declines [19, 20]. Results of cell lines and nude mouse studies showed us the dynamic changes of *APC* and *RASSF1A* methylation on both lung adenocarcinoma cell line (A549) and large cell lung cancer cell line (H460) and indicated that peak methylation increase is also at 24 h after cisplatin administration.

Many studies have shown the high methylation frequency of *APC* and *RASSF1A* in the plasma of lung cancer patients [21–28]. Therefore, we chose the two genes and originated the duplex quantitative methylation specific PCR (qMSP) method to assess their promoter methylation. The feature of this new method is simultaneous detection of both genes’ methylation. Several studies have found differences in the methylation frequencies of *APC* or *RASSF1A* among the particular histological types of lung cancer [29–33]. The reason of these conflicting findings may be the difference in detection sites of the two genes’ promoters. Moreover, we offer both *APC* and/or *RASSF1A* to diminish the influence of histological type that might be caused by using a single gene. Additionally, we compared methylation frequencies of *APC*/*RASSF1A* with other five studies [32, 34–37] (Additional file 3: Table S1). The results showed no significant sample selection bias in the present study. Therefore, our *APC*/*RASSF1A* methylation application for lung cancer prognosis is reliable.

Therefore, we detected the quantitative methylation levels of *APC* and *RASSF1A* before and 24 h after the chemotherapy cycles to evaluate tumor response in lung cancer patients. According to the results, we confirmed our hypothesis that meth_24 h_ > meth_0 h_ is a predictor of sensitivity to cisplatin-based chemotherapy in lung cancer patients. The explanation of this hypothesis is as follows: if the tumor cells are sensitive to chemotherapy drugs, they will be heavily destroyed and lead to a temporary mass release of DNA. Therefore, at 24 h after medication, circulating DNA levels will be higher than that before medication. As indicated in quadrant IV of Fig. 4a, circulating DNA of patients with good drug response was mainly from cancer cells, while circulating DNA of patients with severe toxicity (quadrant II, Fig. 4a) was mainly from healthy cells.

Moreover, Kaplan–Meier analysis and the dynamic methylation monitoring of patients also indicate that patients with increased *APC* or *RASSF1A* methylation levels after cisplatin-based chemotherapy have better outcomes. Additionally, although a patient’s tumor lesion may undergo alterations which are characteristics of remission after effective therapy, the symptoms do not typically present in time. So before we can observe obvious symptoms and signs, most patients (82.4 %) can use *APC* and *RASSF1A* methylation assay after every chemotherapy cycle to predict tumor response. Therefore, methylation detection of plasma at 24 h after medication is a promising earlier and timing indicator than some tumor markers or CT scanning.

At the last part of the training study, we assayed total plasma DNA to monitor toxicity of chemotherapy in ALC patients. This content shows that toxicity is more common in patients with excessively elevated plasma DNA (DNA_24 h_/DNA_0 h_ > 2). Therefore, when combined with *APC*/*RASSF1A* methylation, there were three models:

1. *APC and/or RASSF1A methylation levels increase with DNA*_*24 h*_*/DNA*_*0 h*_ *≤ 2.* This model indicates that patients will benefit from this chemotherapy regimen without severe toxicity. Therefore, to get a better chemotherapy effect, patients can receive higher dosage of chemotherapy drugs while monitoring both total plasma DNA and *APC*/*RASSF1A* methylation levels.
2. *APC and/or RASSF1A methylation levels increase with DNA*_*24 h*_*/DNA*_*0 h*_ *> 2.* In this model, good tumor response and obvious toxicity co-exist. Thus, dosage may need adjustment (usually downward) to avoid severe side effects.
3. *APC and RASSF1A methylation levels never increase no matter how total plasma DNA changes*. In this model, tumor cells are not sensitive to the chemotherapy regimen. Hence, the regimen is not an optimal choice and should be changed immediately.

**Fig. 5:**
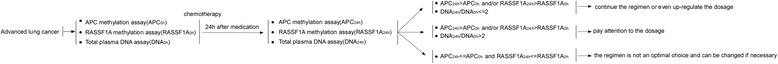
The strategy of using combined *APC*/*RASSF1A* methylation and total plasma DNA to assess chemotherapy response and toxicity and to adjust regimens

Based on the above results of this study, we offer a new strategy to assess the efficiency and toxicity of chemotherapy for ALC (Fig. 5), according to which clinicians can adjust chemotherapy regimens in time.

## Conclusions

We were able to demonstrate that quantitative assay of total plasma DNA and *APC*/*RASSF1A* methylation levels can rapidly and simultaneously predict tumor response and toxicity to cisplatin-based chemotherapy in ALC patients. Especially, the quantitative methylation assay of TSG can show chemosensitivity 24 h after chemotherapy cycles, which is much faster than the imaging-based examinations used in the RECIST guideline. Furthermore, plasma DNA and TSG methylation assays are simple and non-invasive tools, convenient for follow-up and repeated sample collection. The new strategy shown in Fig. 5 may therefore provide a reference or supplement to guidelines in evaluating chemotherapy effects.

## Methods

### Cell culture, MTT assay, and supernatant collection

Human lung adenocarcinoma A549 cells (Shanghai Institutes for Biological Sciences, China) were seeded in triplicate at a density of 1.5 × 10^4^ cells/100 μl in 96-well plates and grown at 37 °C in a humidified atmosphere containing 5 % CO_2_. After 24 h, 100 μl/well cisplatin was added to the cells at three final concentrations (5, 0.5, and 0.05 mg/ml). Cells without cisplatin treatment served as controls. At 0, 6, 12, 24, 48, and 72 h after administration, the culture supernatant was collected. Then, 20 μl MTT (5 mg/ml) was added to each well, and the plate was incubated at 37 °C for another 4 h, after which 10 % HCL-SDS (100 μl) was added to each well. Cell proliferation was determined using a 96-well plate reader to record the absorbance at 570 nm. The inhibition percentage of A549 cell proliferation was calculated using the following formula: inhibition rate = ([absorbance of control well] − [absorbance of administration well])/[absorbance of control well] × 100 %. Data are expressed as mean ± SD of triplicate wells and are representative of at least two independent experiments.

### Nude mouse model of lung adenocarcinoma and blood or tumor tissues collection

Twenty-four nude mice were purchased from Shanghai SLAC Laboratory Animal Company. Eight mice were designated as control group, and two of which died. The other 16 nude mice were used to establish a lung adenocarcinoma model by subcutaneous injection of 2 × 10^6^ A549 cells in the hind legs. When tumor diameters were more than 5 mm after 25 days, the 16 tumor-bearing nude mice were randomly divided into two groups: group 1 consisted of eight tumor-bearing nude mice that were intratumorally injected with 0.2 ml normal saline (NS) containing 8 μg cisplatin per gram of mouse body weight for three consecutive days. Group 2 consisted of eight tumor-bearing nude mice that were intratumorally injected with 0.2 ml NS for three consecutive days.

Blood and tumor tissues of the four groups and controls were collected at 24, 48, and 72 h after administration. To obtain plasma, the blood specimens were centrifuged in two steps at 4 °C (3000 rpm for 10 min and 16,000 rpm for 10 min) [19]. The tumor tissues were prepared by H&E staining.

### Advanced lung cancer patients

**Fig. 6:**
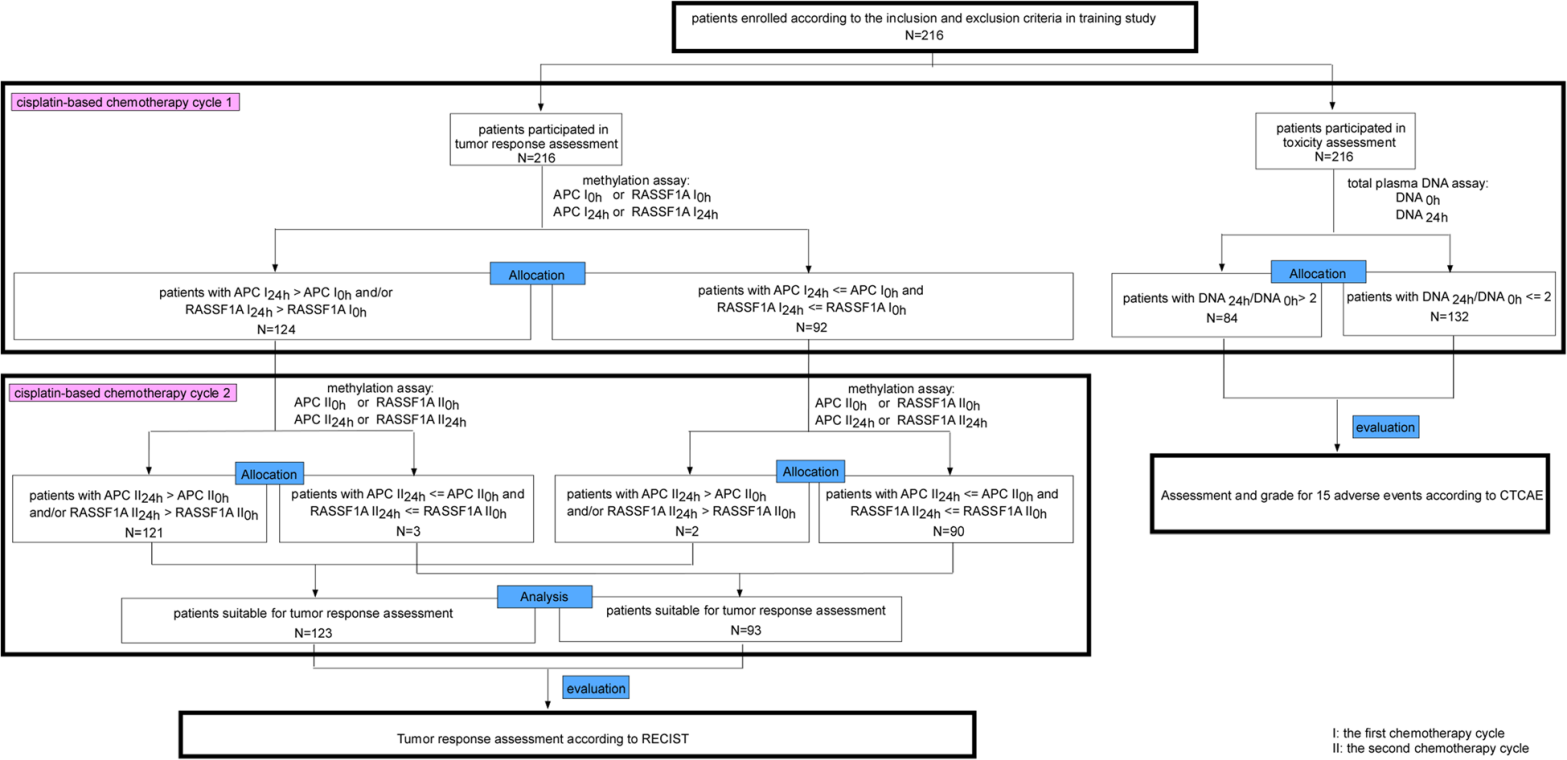
Flow chart of the training study (*n* = 216)

Patients with stages IIIb and IV ALC were recruited from the Department of Oncology of the First Affiliated Hospital of Nanjing Medical University (China) from October 2007 to June 2009. All patients were followed until November 2010. Inclusion criteria were (1) histologically or cytology confirmed lung cancer, (2) newly diagnosed disease according to the staging system of the 2002 American Joint Committee on Cancer (AJCC), (3) an age of 18–80 years, (4) unresectable or metastatic disease, (5) measurable disease, (6) Eastern Cooperative Oncology Group (ECOG) performance status 0–2, (7) life expectancy > 3 months, (8) no contraindication for chemotherapy, and (9) cisplatin-based regimens for two initial cycles. The exclusion criteria were as follows: (1) a history of previous malignant neoplasms or other concomitant malignant diseases; (2) a history of surgery, chemotherapy, and radiotherapy; and (3) refusal to have blood drawn for times needed for this study. All patients gave informed consent prior to specimen collection according to the study, which was reviewed and approved by the Committee on the Ethics of Treatment of Human Subjects of The First Affiliated Hospital of Nanjing Medical University. Two hundred and sixteen patients were included in the training study, and flow chart of which is presented in Fig. 6. Another 100 patients were enrolled in the validation study. The patients’ characteristics are listed in Additional file 4: Table S2.

The blood samples in EDTA-containing tubes, which were used to perform total plasma DNA and methylation assays, were collected from these patients before and 24 h after every chemotherapy cycle and were centrifuged in two steps at 4 °C (3000 rpm for 10 min and 16,000 rpm for 10 min) to obtain plasma.

### Evaluation of chemotherapy efficiency and toxicity

All the patients were followed for at least four chemotherapy cycles. Chemotherapy regimens were IP (irinotecan/cisplatin), DC (docetaxel/cisplatin), and GP (gemcitabine/cisplatin). Chemotherapy efficacy was evaluated by tumor response according to the Response Evaluation Criteria in Solid Tumors (RECIST1.1) [38] 50–60 days after the first cycle. The main evaluation contents were efficient response rate (ERR), tumor markers (detected 2 months after the first cycle), and overall survival (OS). Efficient response included complete response (CR) and partial response (PR). Non-efficient response included stable disease (SD) and progressive disease (PD). Tumor markers were CEA, NSE, and CY21-1, which are the most commonly used markers in lung cancer. Fifteen main toxicities were assessed and graded according to the Common Terminology Criteria for Adverse Events (CTCAE, Version 4.0) after a single chemotherapy cycle.

### DNA extraction from culture supernatant or plasma

DNA was extracted from 200 μl cell culture supernatant or mouse and patient plasma using the BilaTest DNA Kit (Bilatec, Viernheim, Germany), according to the manufacturer’s recommendations. Briefly, 200 μl of specimen was mixed thoroughly with lysis solution, poly(A)RNA/protease solution, and beads/binding buffer, and tubes were placed on a magnet. After discarding the supernatant, the DNA was washed twice with washing solution and then eluted with 40 μl of elution buffer.

### Total plasma DNA assay

Duplex real-time PCR was performed to detect total plasma DNA concentrations, which is described in our previous study [39].

### Duplex qMSP assay of APC and RASSF1A promoter methylation levels

The NCI-H460 cell line was introduced into this study as positive control for methylation of *APC* and *RASSF1A*, as previously described [39]. External standard curves were prepared by serial diluted calibrators and contained 1.5 × 10^5^, 1.5 × 10^4^, 1.5 × 10^3^, and 1.5 × 10^2^ copies/ml of H460 cell DNA using the plasma of a healthy person as solution matrix. The extracted DNA samples (50 μl), including that of the cell culture supernatant, nude mice, patients, and H460 cells, were modified by sodium bisulfate using a CpGenome DNA Modification Kit (Chemicon, USA) based on the principles and procedure previously described by Herman [40]. To fully utilize the trace quantity of modified DNA, we developed a duplex qMSP to measure *APC* and *RASSF1A* simultaneously in the same tube. Each amplification mix (50 μl) contained sample DNA (5 μl) with components supplied in the Takara Ex Taq R-PCR Version 2.1 kit (Takara, Dalian, China). Each reaction contained 10 μl of 5× real-time PCR buffer, 400 μM each deoxynucleotide triphosphate, 2 mM MgCl2, 200 nM each primer, 170 nM each probe, and 2U Takara Ex Taq HS. All the primers and probes used in this protocol were designed by Primer Express Software Version 3.0 and are listed in Additional file 5: Table S3. Amplification and real-time measurement were performed in an Applied Biosystems 7500 Sequence Detector (Applied Biosystems, CA, USA) under the following conditions: 1 min at 95 °C followed by 55 cycles of 5 s at 95 °C and 34 s at 60 °C. Data obtained during 55 cycles of amplification were analyzed. All samples were tested in triplicate. Blood samples pre- and post-chemotherapy from one patient were analyzed simultaneously. Data are expressed as median and interquartile.

### Statistical analysis

All statistical analyses were carried out using Stata9.1 and SPSS13.0 software. Two-way ANOVA tests were used to compare methylation levels between treatment and control groups of A549 cells when three time points were assessed. The methylation rates between tumor-bearing nude mice groups were analyzed using the Fisher’s exact test. Quantitative methylated genes before and after the first chemotherapy cycle was assessed by the Wilcoxon matched-pairs signed-rank test. Differences between groups were analyzed using the Kruskal–Wallis signed-rank test. Survival curves were calculated using the Kaplan–Meier method, and comparisons were performed by the log-rank test. *P* < 0.05 was considered significant.

## Additional files

Additional file 1: Figure S1. Circulating DNA diagram. Total circulating DNA comes from normal cells and tumor cells, whereas aberrant genes hypermethylation usually occur in tumor cells. (TIFF 2509 kb) Additional file 2: Figure S2. Methylation amplification curves for *APC* or *RASSF1A* in A549 cells. (A) Methylation amplification curves for *APC* gene promoter. (B) Methylation amplification curves for *RASSF1A* gene promoter. The Ct values for *APC* and *RASSF1A* gene amplification in A549 cells were close to those of the H460 positive control cells. (TIFF 1393 kb) Additional file 3: Table S1. Methylation frequency of APC and/or RASSF1A in present study and other 5 articles. (DOCX 80 kb) Additional file 4: Table S2. Clinical characteristics of 316 advanced lung cancer patients. (DOCX 18 kb) Additional file 5:Table S3. Primers and probes used in duplex qMSP. (DOCX 13.7 kb)

## Abbreviations

AJCC: American Joint Committee on Cancer
ALC: advanced lung cancer
CEA: carcinoembryonic antigen
CR: complete response
CTCAE: Common Terminology Criteria for Adverse Events
DC: docetaxel and cisplatin
DNA_0 h_: total plasma DNA concentration before chemotherapy treatment
DNA_24 h_: total plasma DNA concentration at 24 h after chemotherapy treatment
ECOG: Eastern Cooperative Oncology Group
ERR: efficient response rate
GP: gemcitabine and cisplatin
IP: irinotecan and cisplatin
meth_0 h_: methylation level before chemotherapy treatment
meth_24 h_: methylation level at 24 h after chemotherapy treatment
NS: normal saline
NSE: neuronal specific enolase
OS: overall survival
PD: progressive disease
PR: partial response
RECIST: Response Evaluation Criteria in Solid Tumors
SD: stable disease
TSG: tumor suppressor genes
