# Supplementary figures and images for: Real-time monitoring efficiency and toxicity of chemotherapy in patients with advanced lung cancer
Source: Clin Epigenetics. 2015 Nov 5;7:119. doi: 10.1186/s13148-015-0150-9 (PMC4635986; doi:10.1186/s13148-015-0150-9)

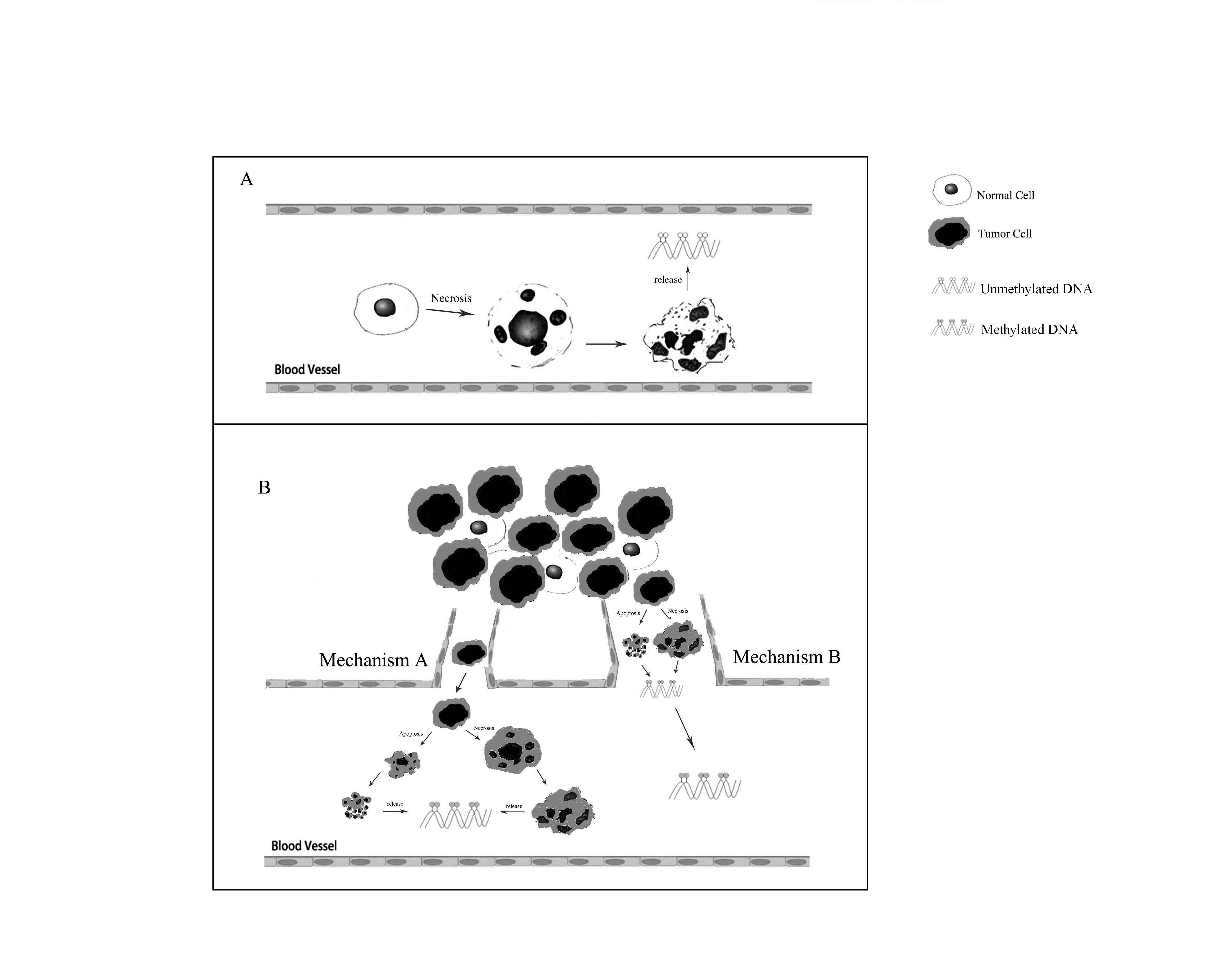

Supplement: Additional file 1: Figure S1. — Circulating DNA diagram. Total circulating DNA comes from normal cells and tumor cells, whereas aberrant genes hypermethylation usually occur in tumor cells. (TIFF 2509 kb) [file 13148_2015_150_MOESM1_ESM.tiff]

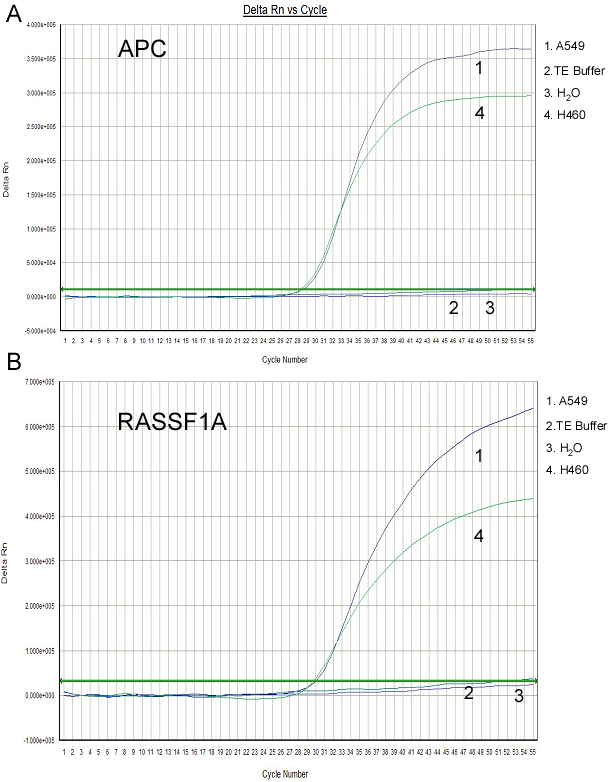

Supplement: Additional file 2: Figure S2. — Methylation amplification curves for APC or RASSF1A in A549 cells. (A) Methylation amplification curves for APC gene promoter. (B) Methylation amplification curves for RASSF1A gene promoter. The Ct values for APC and RASSF1A gene amplification in A549 cells were close to those of the H460 positive control cells. (TIFF 1393 kb) [file 13148_2015_150_MOESM2_ESM.tiff]
