# Supplementary material for: Real-time monitoring efficiency and toxicity of chemotherapy in patients with advanced lung cancer
Source: Clin Epigenetics. 2015 Nov 5;7:119. doi: 10.1186/s13148-015-0150-9 (PMC4635986; doi:10.1186/s13148-015-0150-9)
Supplement: Additional file 3: Table S1. — Methylation frequency of APC and/or RASSF1A in present study and other 5 articles. (DOCX 80 kb) [file 13148_2015_150_MOESM3_ESM.docx]

**Additional file**

**Supplementary Table S1. Methylation frequency of APC and/or RASSF1A in present study and other 5 articles**

| References | Cases | Stage | Specimen | Methylation frequency (%) | |
| --- | --- | --- | --- | --- | --- |
|  |  |  |  | APC | RASSF1A |
| Usadel et al. 2002 [34] | 89 | I-IV | serum/plasma | 47.2 | / |
| Rykova et al. 2004 [35] | 9 | not mentioned | plasma | 33.3 | 44.4 |
| Begum et al. 2011 [36] | 25 | I-IV | serum | 36.0 | 8.0 |
| Zhang et al. 2011 [37] | 110 | I/II | plasma | 47.3 | 36.4 |
| Gao et al. 2015 [32] | 58 | I/II | plasma | 24.1 | 43.1 |
|  | 40 | I/II | serum | 42.5 | 52.5 |
| Our study | 216 | III b-IV | plasma | 31.7 | 43.5 |
