# Supplementary material for: Real-time monitoring efficiency and toxicity of chemotherapy in patients with advanced lung cancer
Source: Clin Epigenetics. 2015 Nov 5;7:119. doi: 10.1186/s13148-015-0150-9 (PMC4635986; doi:10.1186/s13148-015-0150-9)
Supplement: Additional file 4: Table S2. — Clinical characteristics of 316 advanced lung cancer patients. (DOCX 18 kb) [file 13148_2015_150_MOESM4_ESM.docx]

**Supplementary Table S2. Clinical characteristics of 316 advanced lung cancer patients.**

| Characteristics | Training study | | |  | Validation study | | |
| --- | --- | --- | --- | --- | --- | --- | --- |
|  | Median(range) | N | % |  | Median(range) | N | % |
| Age (years) | 62 (45-80) |  |  |  | 63 (48-76) |  |  |
| < median |  | 94 | 43.5 |  |  | 43 | 43.0 |
| ≥median |  | 122 | 56.5 |  |  | 57 | 57.0 |
| Gender |  |  |  |  |  |  |  |
| Male |  | 121 | 56.0 |  |  | 73 | 73.0 |
| Female |  | 95 | 44.0 |  |  | 27 | 27.0 |
| Smoke |  |  |  |  |  |  |  |
| Smoker |  | 85 | 39.4 |  |  | 64 | 64.0 |
| Non smoker |  | 131 | 60.6 |  |  | 36 | 36.0 |
| AJCC Stage |  |  |  |  |  |  |  |
| Ⅲb |  | 38 | 17.6 |  |  | 23 | 23.0 |
| Ⅳ |  | 178 | 82.4 |  |  | 77 | 77.0 |
| Pathological classification |  |  |  |  |  |  |  |
| Adenocarcinoma |  | 94 | 43.5 |  |  | 65 | 65.0 |
| Squamous carcinoma |  | 56 | 25.9 |  |  | 17 | 17.0 |
| other NSCLC |  | 28 | 13.0 |  |  | 7 | 7.0 |
| SCLC |  | 38 | 17.6 |  |  | 11 | 11.0 |
| Tumor response(after 2 cycles) |  |  |  |  |  |  |  |
| CR |  | 0 | 0.0 |  |  | 2 | 2.0 |
| PR |  | 101 | 46.8 |  |  | 62 | 62.0 |
| SD |  | 46 | 21.3 |  |  | 27 | 27.0 |
| PD |  | 69 | 31.9 |  |  | 9 | 9.0 |
| Survival time(months) | 15 (4-36) |  |  |  | 17 (6-28) |  |  |
| < median |  | 103 | 47.7 |  |  | 44 | 44.0 |
| ≥median |  | 113 | 52.3 |  |  | 56 | 56.0 |
