# Supplementary material for: Real-time monitoring efficiency and toxicity of chemotherapy in patients with advanced lung cancer
Source: Clin Epigenetics. 2015 Nov 5;7:119. doi: 10.1186/s13148-015-0150-9 (PMC4635986; doi:10.1186/s13148-015-0150-9)
Supplement: Additional file 5:Table S3. — Primers and probes used in duplex qMSP. (DOCX 13.7 kb) [file 13148_2015_150_MOESM5_ESM.docx]

**Supplementary Table S3. Primers and probes used in duplex qMSP.**

| Genes | Sequence | Amplicon (bp) |
| --- | --- | --- |
| APC | Forward: 5’-GGGTCGCGAGGGTATATTTTC-3’  Reverse: 5’-CCGACCCGCACTCCG-3’  Probe: 5’(JOE)-CCCGCCCAACCGCACAACCT(Eclipse)-3’ | 97 |
| RASSF1A | Forward: 5’-GTCGTTGTGGTCGTTCGG-3’  Reverse: 5’-GAAACTAAACGCGCTCTCG-3’  Probe: 5’(FAM)-CCTTACCCTTCCTTCCCTCCTTCGT(Eclipse)-3’ | 95 |

JOE = 2,7-dimethyl-4,5-dichlorine-6-carboxyfluorescein

FAM= 6-carboxyfluorescein

Eclipse is a non-fluorescent quencher.
